# Daily administration of yokukansan and keishito prevents social isolation-induced behavioral abnormalities and down-regulation of phosphorylation of neuroplasticity-related signaling molecules in mice

**DOI:** 10.1186/s12906-017-1710-7

**Published:** 2017-04-04

**Authors:** Hironori Fujiwara, Yaoyu Han, Ken Ebihara, Suresh Awale, Ryota Araki, Takeshi Yabe, Kinzo Matsumoto

**Affiliations:** 1 Division of Medicinal Pharmacology, Institute of Natural Medicine, University of Toyama, Toyama, Japan; 2 Division of Natural Drug Discovery, Institute of Natural Medicine, University of Toyama, Toyama, Japan; 3 Laboratory of Functional Biomolecules and Chemical Pharmacology, Faculty of pharmaceutical sciences, Setsunan University, Osaka, Japan

**Keywords:** Attention deficit hyperactivity disorder, Social isolation, Yokukansan, Keishito, Kampo formulae, Developmental disorders

## Abstract

**Background:**

Our previous studies demonstrated that post-weaning social isolation (ISO) in mice induces behavior abnormalities such as deficits of sociability- and attention-like behaviors. These deficits can be attenuated by methylphenidate (MPH), a drug used for attention deficit hyperactivity disorder (ADHD), suggesting that ISO mice offer a potential animal model of comorbid developmental disorder with ADHD and autism spectrum disorder symptoms. This study investigated the effects of Kampo formulae, yokukansan (YKS) and keishito (KST), on the neuropsychiatric symptoms of ISO mice to clarify the therapeutic or preventive/delaying potential of these formulae for the treatment of neurodevelopmental disorders.

**Methods:**

Three-to-4-week old male ICR mice were socially isolated during an experimental period and YKS and KST (1523.6 and 2031.8 mg/kg, p.o.) was administered starting from week 2 and week 0 after starting ISO for the analysis of their therapeutic and preventive/delaying potentials, respectively. Sociability, attention-related behavior and fear memory were elucidated by a 3 chamber test, a water-finding test and fear conditioning test, respectively. Moreover, the phosphorylation of neuroplasticity-related signaling molecules in mice hippocampus was analyzed using western blotting.

**Results:**

In a therapeutic procedure, YKS ameliorated ISO-induced impairments of attention-like behavior and context-dependent fear memory, but not of sociability, whereas KST had no beneficial effects in ISO mice. In experiments to analyze the preventive/delaying potentials of these treatments, both YKS and KST improved sociability, attention, and context-dependent fear memory deficits. The improvement of sociability in mice by YKS and KST was not inhibited by a dopamine D_1_ receptor antagonist, suggesting that YKS and KST improved the ISO-induced sociability deficit by other mechanisms besides activation of the dopaminergic system. On the other hand, the beneficial effects of YKS and KST on attention-like behavior were inhibited by a muscarinic antagonist, suggesting that YKS and KST ameliorated ISO-induced attention-like behavior through a cholinergic mechanism. Moreover, the phosphorylated forms of CaMKII and CREB were down-regulated by ISO stress and restored by YKS and KST administration.

**Conclusions:**

These findings suggest that YKS and KST may be useful for the improvement of neurodevelopmental disorders.

**Electronic supplementary material:**

The online version of this article (doi:10.1186/s12906-017-1710-7) contains supplementary material, which is available to authorized users.

## Background

Attention deficit hyperactivity disorder (ADHD) is a common childhood and adolescent neurodevelopmental disorder characterized by core symptoms such as hyperactivity, inattention, and impulsivity. Moreover, ADHD have a high incidence rate of psychiatric comorbidity disorder, which is accompanied by other developmental and behavioral symptoms including sociability and leaning deficit [1]. The symptoms of ADHD usually decline with growth; however, it is a serious problem that about two-thirds of patients are not completely cured even in adulthood [2]. It has been suggested that dysfunctions of dopaminergic systems in the brain contribute to the pathogenetic basis of ADHD, because the amount of dopamine is reduced in the striatum and prefrontal cortex of ADHD patients [3], and methylphenidate (MPH), a dopamine transporter inhibitor, reverses the down-regulation of dopamine in these areas and improves abnormal behavior in ADHD patients [4]. Moreover, serotonergic and cholinergic dysfunctions also contribute to the development of ADHD [5, 6]. There are genetic and environmental factors for the onset of ADHD; the former includes various genetic variations such as in the dopamine transporter [7–9] and receptors [10, 11], serotonin 5-HT_2A_ receptor [12], and N-methyl-D-aspartate glutamate receptor subunit [13]. Moreover, environmental factors appear to alter the epigenetic regulation of the disorder [14].

We have previously demonstrated that post-weaning social isolation (ISO) rearing of rodents causes behavioral abnormalities similar to those of ADHD patients such as attention deficit-like behaviors, increased aggressive responses, hyperactivity and fear memory deficits [15–18] and that ISO-induced attention deficit-like behaviors were reversed by MPH [18]. Moreover, our neurochemical studies have suggested that ISO-induced fear memory deficits are in part mediated by a decrease in the expression level of phosphorylated forms of neuroplasticity-related signaling molecules, such as CaMKII and CREB, since the administration of tacrine, an acetylcholinesterase inhibitor, reverses the down-regulated expression of these phosphorylated forms in the hippocampus by mechanisms relevant to improvement of fear memory deficits in ISO mice [19, 20]. Moreover, we found that ISO mice also showed impairment of sociability [20]. On the other hand, clinical evidence indicates that deficits in sociability are one of the core symptoms of autism spectrum disorder (ASD) and that a considerable number of patients with ASD exhibit comorbid symptoms of ADHD [21, 22]. Taken together, our previous findings suggest that ISO mice provide a putative animal model of developmental disorders including the comorbidity of ADHD and ASD [23, 24] to explore drugs with therapeutic and/or preventive potentials for the disorders.

Traditional herbal medicines such as Kampo and Chinese medicines have been used to treat the symptoms of many diseases. Moreover, clinical studies in patients with psychological disorders have shown that some traditional herbal medicines can ameliorate the positive and negative syndrome scale of schizophrenia, and the symptoms of inattention, hyperactivity, and impulsivity in children and adolescents [25, 26]. These studies suggest that novel agents effective for treating neuropsychological disorders may be found in traditional herbal remedies. Recently, Iwasaki et al. have reported that yokukansan (YKS), a traditional herbal medicine that has long been used for patients with neurosis, insomnia, and irritability in children, can improve the behavioral and psychological symptoms of dementia patients such as hallucinations, agitation, and aggression in patients with Alzheimer’s disease and other forms of senile dementia [27, 28]. Moreover, it has been reported that YKS also improves not only memory disturbances but also abnormal social interaction, such as increased aggressive behavior and decreased social behavior, in amyloid precursor protein transgenic mice [29], suggesting that YKS can exert a beneficial effect on symptoms of several neuropsychological disorders. These clinical and experimental findings prompted us to investigate whether YKS has therapeutic and preventive/delaying potentials for neurodevelopmental disorders. In the present study, we employed ISO mice as a putative animal model of developmental disorders with comorbid features of ADHD and ASD and elucidated the pharmacological effects of YKS on ISO-induced behavioral abnormalities and neurochemical alterations in the brain. We also used keishito (KST) as a reference Kampo prescription because the clinical use of KST is different from that of YKS. Indeed, KST is prescribed for the initial symptoms of a cold with a headache and fever in children. In this study, we compared the effects of YKS with those of KST to elucidate whether these prescriptions for children exert different pharmacological effects in ISO mice.

## Methods

### Preparation and chemical profiling of YKS and KST

The following medicinal herbs, which conformed to the Japanese Pharmacopiea XVI, were purchased from Tsumura Co. (Tokyo, Japan) and Tochimoto tenkai-do (Osaka, Japan) to prepare YKS and KST. YKS was extracted from a mixture of 4.0 parts *Atractylodis lanceae Rhizoma*, 4.0 parts *Poria*, 3.0 parts *Cnidii Rhizoma*, 3.0 parts *Uncariae Uncis* cum *Ramulus*, 3.0 parts *Angelicae Radix*, 2.0 parts *Bupleuri Radix*, and 1.5 parts *Glycyrrhizae Radix*. KST was extracted from a mixture of 4.0 parts *Cinnamomi Cortex*, 4.0 parts *Paeoniae Radix*, 4.0 parts *Zizyphi Fructus*, 2.0 parts *Glycyrrhizae Radix*, and 1.5 parts *Zingiberis Rhizoma*. The yields of YKS and KST extract were 17.4% and 18.9%, respectively. The chemical constituents of YKS and KST were analyzed by a Shimadzu LC-IT-TOF mass spectrometer equipped with an ESI interface. The ESI parameters were as follows: source voltage, +4.5 kV (positive ion mode) or −3.5 kV (negative ion mode); capillary temperature, 200 °C; and nebulizer gas, 1.5 l/min. A Waters Atlantis T3 column (2.1 mm × 100 mm) was maintained at 40 °C. The mobile phase was a binary eluent of (A) 5 mM ammonium acetate solution and (B) acetonitrile under the following gradient conditions: 0–50 min; linear gradient from 10% to 100% B, and 50–60 min; isocratic at 100% B. The flow rate was 0.2 ml/min. Mass spectrometry data obtained from YKS and KST have been stored in the Wakan-Yaku DataBase system, Institute of Natural Medicine, University of Toyama (YKS: http://dentomed.u-toyama.ac.jp/en/information_on_experimental_kampo_extracts/yokukansan-2016-KM-extract/EXP081002; KST: http://dentomed.u-toyama.ac.jp/en/information_on_experimental_kampo_extracts/keishito-2016-KM-extract/EXP010002), and can be browsed according to the manual in additional file (Additional file 1). Voucher specimens (YKS: No. 20000005, KST: No. 20000006) had been deposited at our institute.

### Animals

**Fig. 1:**
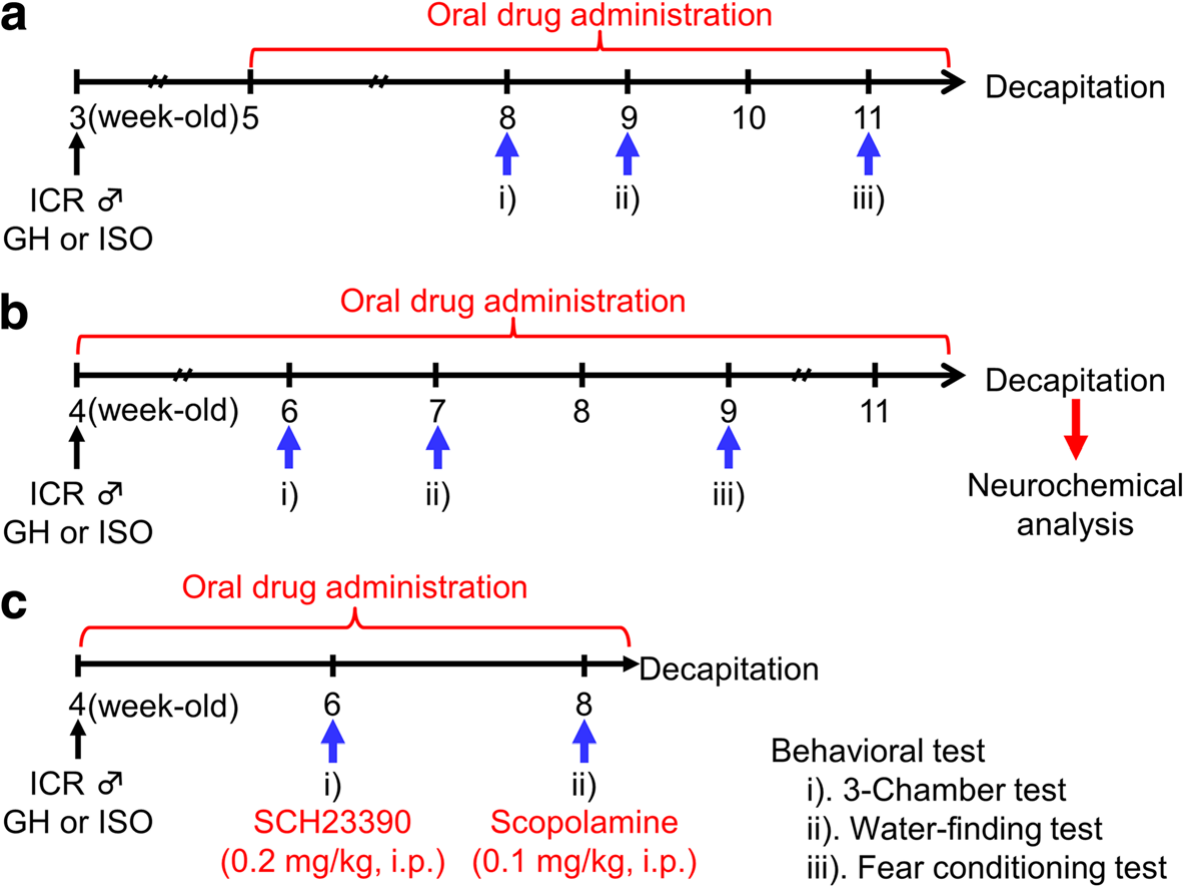
Experimental schedule. **a** The therapeutic procedure: ISO and oral drug administration were started at the age of 3 and 5 week-old and continued during the experimental period. The 3-chamber sociability test (i), water-finding test (ii), and fear conditioning test (iii) were conducted at the age of 8, 9, and 11 week-old, respectively. **b** The preventive/delaying procedure: ISO and oral drug administration were started from at the age of 4 week-old and continued during the experimental period. The 3-chamber sociability test (i), water-finding test (ii), and fear conditioning test (iii) were conducted at the age of 6, 7, and 9 week-old, respectively. **c** The pharmacological analysis of preventive/delaying effect: ISO and oral drug administration were started at the age of 4 week-old and continued during the experimental period. The 3-chamber sociability test (i) and water-finding test (ii) were conducted at the age of 6 and 8 week-old, respectively. Animals were decapitated at 12 (**a** and **b**) or 8 (**c**) week-old, after completing all of the behavioral experiments

The study was conducted according to the experimental schedule described in Fig. 1 Male ICR mice at the age of 3 or 4 weeks were obtained from Japan SLC (Shizuoka, Japan). Animals were housed in groups (group housing: GH) of 4–5 mice/cage (24 × 17 × 12 cm) or socially isolated (ISO) in the same size cages, as previously reported [20]. ISO mice were raised alone until decapitation. The housing room was maintained at 24 ± 1 °C with 65% humidity and a 12-h light-dark cycle (lights on: 07:30–19:30). Food and water were given ad libitum. All animal research procedures used in the present study were in accordance with the Guiding Principles for the Care and Use of Animals (NIH Publications No. 80–23, revised in 1996). The present study was also approved by the Institutional Animal Use and Care Committee of the University of Toyama.

### Drug treatment

The administration of YKS (1523.6 mg/kg/day) or KST (2031.8 mg/kg/day) at doses approximately 30 times more than the typical daily doses for human therapy was started from week 2 after starting ISO (therapeutic procedure; Fig. 1a), a period required to cause behavioral abnormalities in our previous study [18], or week 0 (preventive/delaying procedure; Fig. 1b). These doses were in ranges of other drugs, including herbal medicines, which have been used in the studies of animal models reported by our and other research groups [29, 30]. To reduce stress other than ISO as much as possible, ISO animals were given daily YKS and KST extracts or water via a drinking nozzle. GH group was given water by the same way. On the basis of average daily water intake which was calculated in advance, the concentration of YKS and KST extracts were adjusted to give the daily dose of each extract. We confirmed daily during an experimental period that the test drug solutions were completely taken by each animal. SCH-23390 (dopamine D_1_ receptor antagonist; 0.2 mg/kg) and scopolamine (muscarinic receptor antagonist, 0.1 mg/kg), at doses that were enough to reverse the MPH-induced amelioration of behavior deficits in ISO animals [20], were dissolved in saline. Each antagonist was injected intraperitoneally 1 h before the 3-chamber test or water-finding test, as shown Fig. 1c.

## Behavioral analysis

### 3-chamber test

The sociability of mice was assessed using the 3-chamber test as previously reported by Hanks et al. 2 or 5 weeks after starting ISO [31]. The equipment included an open field (67 cm × 14 cm × 21 cm) with a gray walls and black floor, and was divided into 3 equal chambers by 2 clear gates. Two clear cylindrical cages (diameter: 10 cm and height: 12 cm) with black lids were placed in the left and right chambers of the apparatus. In the training trial, each mouse was placed in the center arena of the equipment, and allowed to explore the arena freely for 5 min. The time the mouse spent near the 2 cylindrical cages (around 2 cm) was measured. The arena and cylindrical cages were cleaned using 70% ethanol between trials to prevent selectivity of stimuli due to olfactory cues. The test trials were performed 5 min after the training trial. An unfamiliar mouse was put in one of the cages, while the other cage remained empty. The total time spent near the 2 cylindrical cages was also measured and analyzed automatically using the Smart^®^ system (PanLab,S.L., Barcelona, Spain). Recognition index consisted of dividing empty exploration time (E) or stranger exploration time (S) by the total exploration time (E or S/E + S).

### Water-finding test

A water-finding test was performed 3, 4 or 6 weeks after starting ISO according to the protocol of Ouchi et al. [18]. The equipment consisted of an open field (30 cm × 30 cm × 30 cm) which was connected to the small box (10 cm × 10 cm × 10 cm). A drinking nozzle was set on the center of the small box ceiling with its end 5 and 7 cm above the floor in the training and test trials, respectively. The training and test trials were conducted on day 1 and day 2, respectively. In the training trial, each mouse was placed in 1 corner of the open field. The mice were allowed to explore the equipment for 5 min. Mice that couldn’t find the drinking nozzle during a 5-min observation period were excluded from the test trials. Mice were deprived of water for 24 h after the training trial. In the test trials, the animals were again placed individually into the equipment and the latency for drinking water was measured for each animal.

### Fear conditioning test

A fear conditioning test was performed at 5 or 8 weeks after starting ISO in accordance with the protocol of Okada et al. with minor modifications [20]. Briefly, the equipment for fear conditioning consisted of a clear acrylic chamber (30 cm × 30 cm × 30 cm) and a stainless-steel grid floor equipped with an electric shock generator/scrambler SGS-002^®^, CS Controller CSS-001^®^, and Cycle Timer CMT^®^ (Muromachi Kikai. Co. Ltd., Tokyo, Japan). The equipment was placed in a soundproof observation box (MC-050/CM, Muromachi Kikai, Co. Ltd., Tokyo, Japan) through which an auditory tone (Sonalert^®^, Mallory Sonalert Products Inc., Indianapolis, IN, USA) was delivered to the animal. In the training trial, animals were placed individually into the chamber and allowed to explore freely for 3 min. They then received an acoustic tone (2.9 kHz, 20 s, 80 dB) that co-terminated with electric foot shocks (0.8 mA, 2 s). The tone-foot shock pairing was repeated 5 times with 1-min intervals. One minute after the final foot shock delivery, the mice were returned to their home cage. Mice that did not respond to the foot shocks were excluded from the test trials in accordance with the method of Bontekoe et al. [32]. Contextual fear memories were measured 24 h after the training trial. In the contextual memory test, mice were placed in the same chamber to provide contextual stimuli and allowed to move freely for 6 min. One minute after placing the animal in the chamber, freezing behavior during a 5-min period was recorded as an index of context-dependent fear memory. Animal behavior was video-recorded and analyzed automatically using the Smart^®^ system. Freezing was defined as the absence of any movement, except for those related to respiration, and analyzed as a state with a movement speed no greater than 0.05 cm^2^/s [18, 20].

### Western blotting analysis of neuroplasticity-related signaling molecules in hippocampal tissues

The phosphorylation of CaMKII and CREB was analyzed using western blotting as previously described [20]. Briefly, we randomly selected four mice from each group. The hippocampus was obtained from each mouse and homogenized in protein lysis buffer (50 mM Tris (pH 7.4), 150 mM sodium chloride, 0.5% sodium deoxycholate, 1%(*v*/v) NP-40, 0.1%(*v*/v) sodium dodecyl sulfate (SDS), 150 mM sodium fluoride, 8.12 μg/ml aprotinin, 2 mM sodium orthovanadate, 10 μg/ml leupeptin, and 2 mM phenylmethylsulfonyl fluoride) using TissueLyser® (Qiagen, Osaka, Japan), and centrifuged at 1500×g at 4 °C for 3 min. The supernatant was used in the experiments. Protein concentrations of the samples were determined using a BCA™ protein assay kit (Thermo Scientific, Rockford, IL, USA). Protein extracts containing 10 μg proteins were applied to SDS-polyacrylamide gels and electrophoresed. Separated proteins were transferred to a polyvinylidene difluoride membrane (Immuno-Blot® membrane, Bio-Rad Laboratories, Hercules, CA, USA). Blots were blocked with 2.5% bovine serum albumin in 0.1% Tween 20 containing Tris-buffered saline (TBS-T), and probed with primary antibodies described below using a 1:1000 dilution at 4 °C overnight, followed by incubation with anti-rabbit or anti-mouse IgG antibody HRP-linked in a dilution of 1:2000 at room temperature for 2 h. After washing in TBS-T, chemiluminescence was detected using Immobilon™ Western Chemiluminescent HRP Substrate (Millipore, Billerica, MA, USA). Immunoreactive bands were visualized and analyzed with ImageQuant LAS-4000 and ImageQuant TL^®^ (GE Healthcare Japan, Tokyo, Japan).

The primary antibodies used were: anti-CaMKIIa mouse monoclonal antibody, anti-phospho-CaMKII (p-CaMKII) (pThr286) rabbit polyclonal antibody, anti-CREB (48H2) rabbit monoclonal antibody, and anti-phospho-CREB (p-CREB) (pSer133) rabbit monoclonal antibody.

### Statistics

Data are expressed as the mean ± S.E.M. Data obtained in 3-chamber test were analyzed by the paired t-test for two groups according to previous study [20]. Data obtained in other behavior test and neurochemical studies were analyzed by Student’s t-test for two groups or a one-way ANOVA followed by the Student–Newman–Keuls test for multiple comparisons. *P* values of <0.05 were considered to be significant. The analysis was conducted using SigmaPlot^®^ ver 12.0 software (SYSTAT Software Inc., Richmond, CA, USA).

## Results

### YKS and KST prevented ISO-induced sociability deficits via a dopaminergic neuron-independent mechanism

**Fig. 2:**
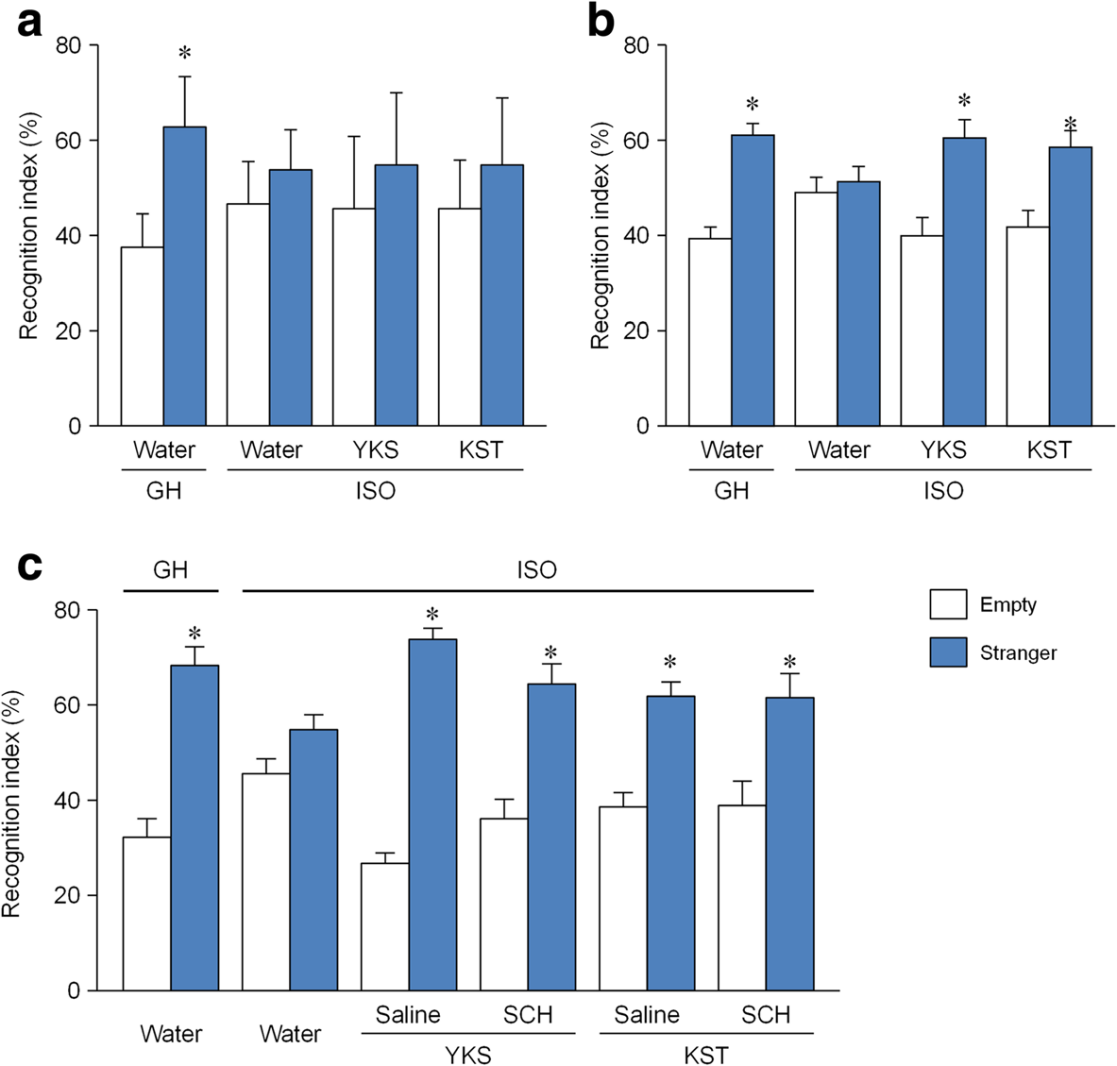
The effects of YKS and KST on ISO-induced sociability deficits in mice. The time a mouse spent near the stranger and empty chambers was measured in the therapeutic procedure (**a**), preventive/delaying procedure (**b**) and pharmacological analysis of preventive/delaying effect (**c**). YKS and KST were administered orally at doses of 1523.6 and 2031.8 mg/kg/day, respectively, for 2–3 weeks before the test, and SCH-23390 (SCH: 0.2 mg/kg) was injected intraperitoneally 1 h before test. The number of each group is 24 (GH-water), 26 (ISO-water), 12 (ISO-YKS) and 12 (ISO-KST) in (**a**), 12, 12, 12 and 12 in (**b**), and 12, 12, 12, 12, 12 (ISO-YKS-SCH) and 11 (ISO-KST-SCH) in (**c**). Recognition index consisted of dividing empty exploration time (*E*) or stranger exploration time (*S*) by the total exploration time (*E* or *S/E* + *S*). Each data column represents the mean ± S.E.M. *p < 0.05 vs. the time a mouse spent near the empty chamber

The 3-chamber test was performed to determine the effects of YKS and KST on ISO-induced sociability deficits. In the experiments to analyze the therapeutic potentials of YKS and KST, GH mice made more approaches to the stranger cage with the naïve mouse and spent more time exploring this chamber than the empty chamber (*t* = −4.653, df = 23, *p* < 0.01). However, in water-treated ISO mice, no significant difference in the time spent exploring was found between the empty and stranger chambers (*t* = −1.029, df = 25, *p* = 0.313), suggesting an impairment of social interaction behavior. Neither YKS nor KST had any effect on the sociability deficit in ISO mice (YKS: *t* = −1.488, df = 11, *p* = 0.165; KST: *t* = −1.130, df = 11, *p* = 0.283) (Fig. 2a). In the experiments conducted to test the preventive or delaying effects of Kampo medicines, GH and ISO mice also showed the same performance as that observed in the experiments to analyze the therapeutic potentials of the test drugs (GH: *t* = −4.452, df = 11, *p* < 0.01; ISO: *t* = −0.360, df = 11, *p* = 0.725). Interestingly, the administration of YKS and KST significantly improved the sociability deficits in ISO mice (YKS: *t* = −2.684, df = 11, *p* < 0.05; KST: *t* = −2.432, df = 11, *P* < 0.05) (Fig. 2b).

Next, we examined the effect of SCH-23390, a dopamine D_1_ receptor antagonist, on the improvement of sociability by YKS and KST. ISO mice were given SCH22390 at 0.2 mg/kg. This antagonist did not affect the improvement effect by YKS and KST (YKS: *t* = −10.165, df = 11, *p* < 0.01; YKS + SCH: *t* = −3.365, df = 11, *p* < 0.01; KST: *t* = −3.894, df = 11, *p* < 0.01; KST + SCH: *t* = −2.217, df = 10, *p* < 0.05) (Fig. 2c).

### YKS and KST improved ISO stress-induced latent learning performance deficit via a cholinergic mechanism in the water-finding test

**Fig. 3:**
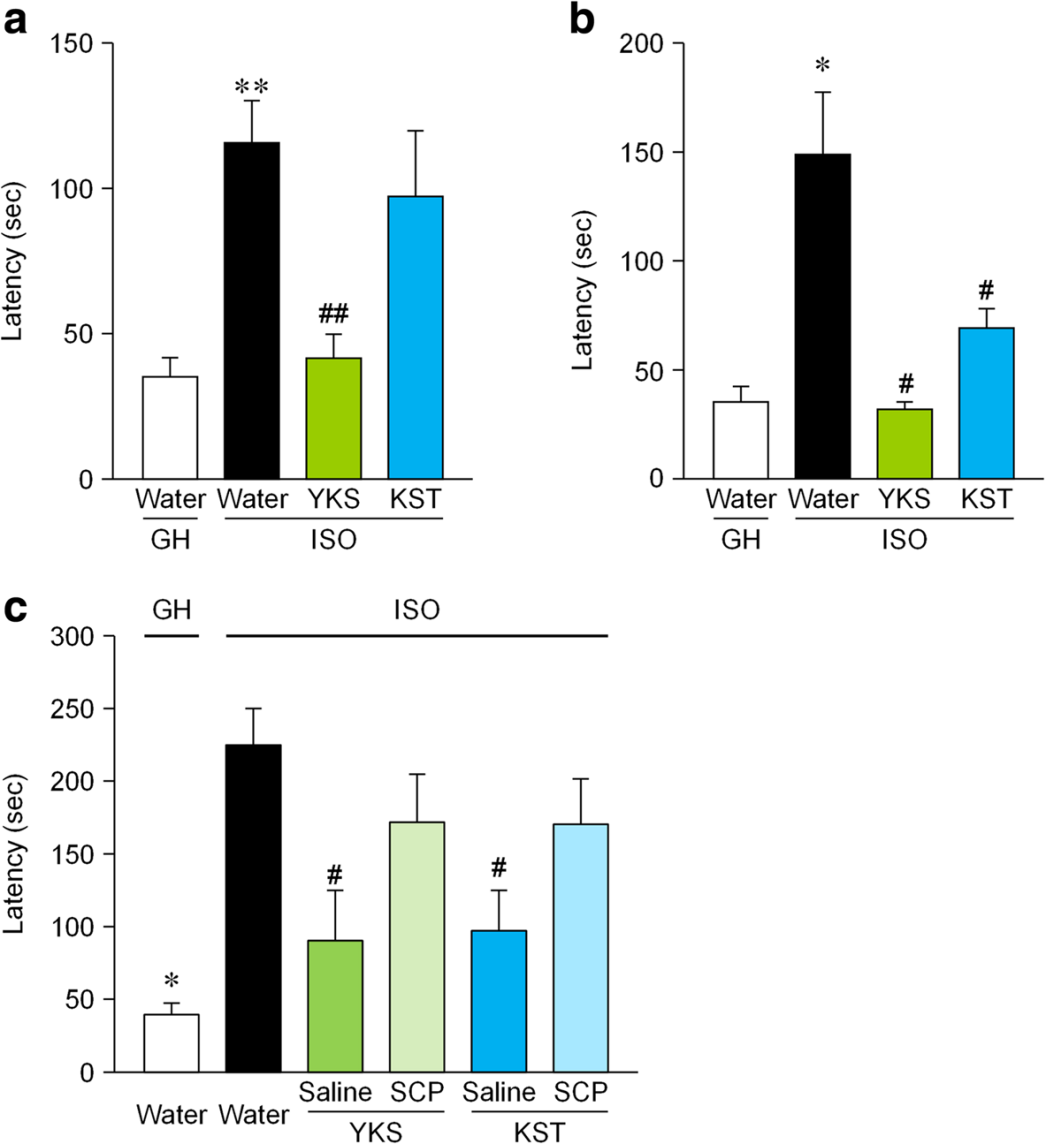
The effect of YKS and KST on ISO-induced attention deficit-like behavior in mice. The latency of each animal was recorded in the therapeutic (**a**) and preventive/delaying procedures (**b**) and pharmacological analysis of preventive/delaying effect (**c**). YKS and KST were administered orally at doses of 1523.6 and 2031.8 mg/kg/day, respectively, for 3–4 weeks before the test, and scoplamine (SCP: 0.1 mg/kg) was injected intraperitoneally 1 h before test. The number of each group is 23 (GH-water), 26 (ISO-water), 12 (ISO-YKS) and 12 (ISO-KST) in (**a**), 12, 12, 12 and 12 in (**b**), and 9, 11, 8, 8, 12 (ISO-YKS-SCP) and 11 (ISO-KST-SCP) in (**c**). Each data column represents the mean ± S.E.M. *p < 0.05, **p < 0.01 compared with GH mice. ^#^ *p* < 0.05, ^##^ *p* < 0.01 compared with the saline-administered ISO group

The effects of YKS and KST on ISO-induced spatial attention deficit were examined by the water-finding test. ISO stress significantly increased the drinking latency of mice compared to that of GH mice in therapeutic (*t* = −4.899, df = 47, *p* < 0.01) and preventive/delaying procedures (*t* = −3.685, df = 22, *p* < 0.01), suggesting a ISO-induced spatial attention deficit. In the therapeutic procedure, the administration of YKS significantly shortened the ISO-increased latency time [F(2, 47) = 5.073, *P* < 0.01], whereas KST had no effect [F(2, 47) = 5.073, *P* = 0.429] (Fig. 3a). However, both YKS and KST ameliorated the ISO-induced spatial attention deficit in the protective procedures [F(2, 33) = 9.940, YKS and KST: *P* < 0.01] (Fig. 3b). Moreover, the drinking latency of YKS- and KST-treated ISO mice decreased after pretreatment with scopolamine at 0.1 mg/kg [F(4, 45) = 2.912, YKS: *p* < 0.05] (Fig. 3c).

### YKS and KST restore the ISO stress-induced context-dependent fear memory deficit in the fear conditioning test

**Fig. 4:**
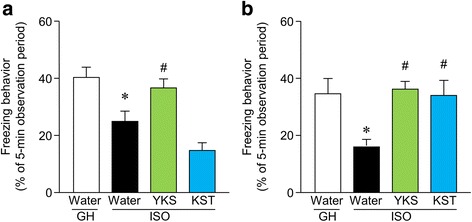
The effect of YKS and KST on ISO-induced long-term fear memory deficits in mice. The Freezing time of contextual experimental condition was recorded in the therapeutic (**a**) and preventive/delaying procedures (**b**). YKS and KST were administered orally at doses of 1523.6 and 2031.8 mg/kg/day for 5–6 weeks before the test. The number of each group is 22 (GH-water), 23 (ISO-water), 12 (ISO-YKS) and 12 (ISO-KST) in (**a**), and 11, 11, 12 and 11 in (**b**). Each data column represents the mean ± S.E.M. *P < 0.05 vs. the GH group. ^#^ *p* < 0.05 compared with the water-administered ISO group

We performed a fear conditioning test in order to determine the effects of YKS and KST on the long-term fear memory deficit in ISO mice. In the therapeutic procedure, the freezing time of ISO mice in the contextual memory test was significantly decreased compared to GH mice (*t* = 3.067, df = 43, *p* < 0.01). YKS extended the freezing time of ISO mice significantly in the contextual cue test [F(2, 44) = 7.707, *P* < 0.05]. On the other hand, KST failed to improve context-dependent fear memories (Fig. 4a). However, in the preventive/delaying procedure, both YKS and KST increased the freezing time of ISO mice in the contextual cue test [F(2, 32) = 6.768, YKS: *P* < 0.01; KST: *P* < 0.01] (Fig. 4b).

### YKS and KST reversed the ISO-induced down-regulation of the phosphorylation of neuroplasticity-related signaling molecules in the hippocampus

**Fig. 5:**
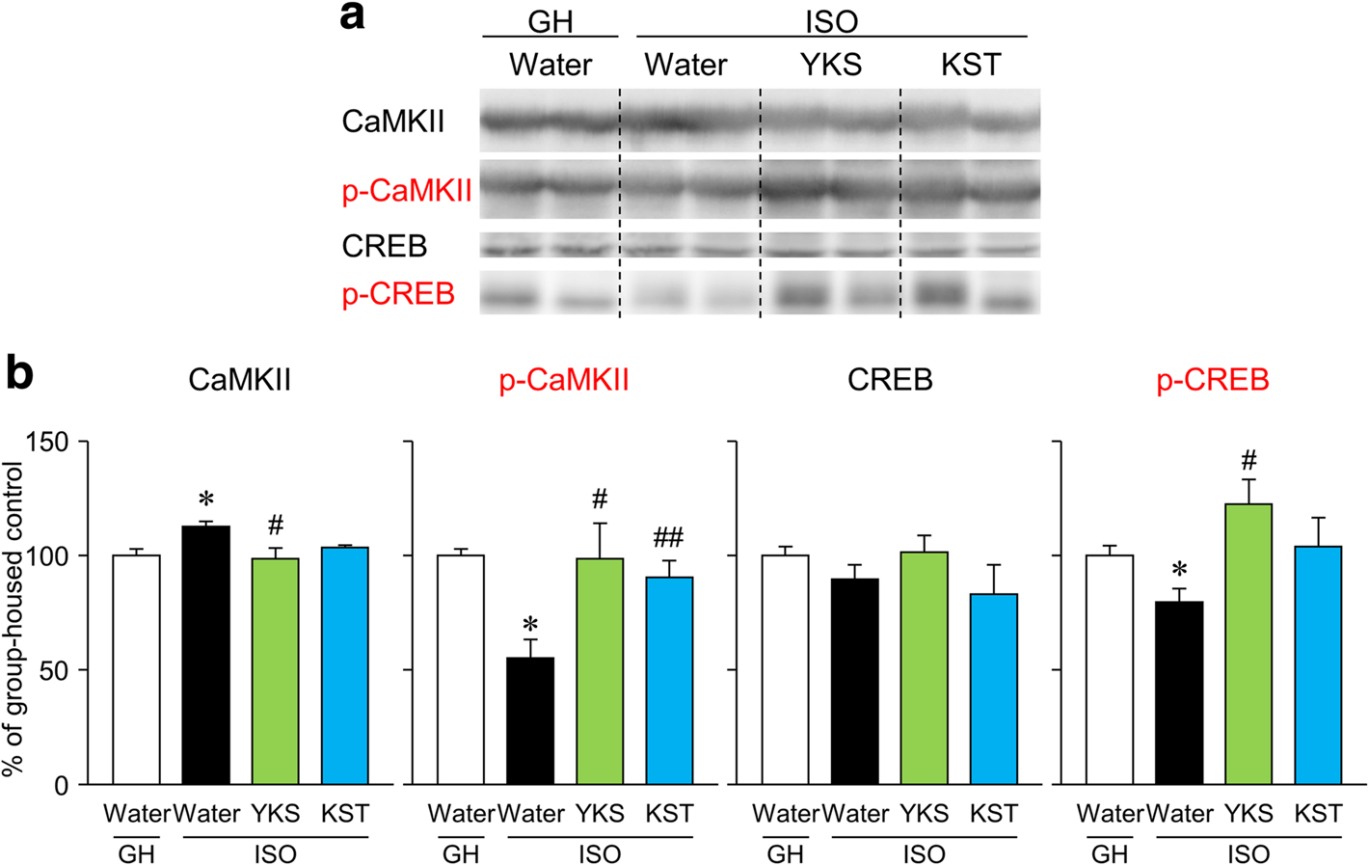
Effects of YKS and KST on ISO-induced down-regulated CaMKII and CREB phosphorylation in the hippocampus. **a** Experiments were conducted after completing the fear conditioning test. Each animal was decapitated, and the hippocampi were dissected out for neurochemical studies. Typical photos indicate the levels of the phosphorylated form of CaMKII and CREB in the hippocampus obtained from GH and ISO treated with water or YKS and KST. The two columns of samples for each group are biological replicates. **b** Quantitative comparisons of the levels of each protein in the hippocampus. Each data column represents the mean ± S.E.M. obtained from 4 brain samples. *P < 0.05 vs. the GH group. #*P* < 0.05, ##*P* < 0.01 vs. the saline-treated ISO group

After the preventive/delaying procedures, we confirmed the effect of ISO on the phosphorylated forms of CaMKII and CREB, neuroplasticity-related signaling molecules, in the hippocampus sections (Fig. 5). ISO for 7 weeks significantly reduced the phosphorylated forms of CaMKII and CREB in the hippocampus (p-CaMKII: *t* = 5.249, df = 6, *p* < 0.01; p-CREB: *t* = 2.756, df = 6, *p* < 0.05). The administration of YKS and KST significantly restored the phosphorylation level of CaMKII in the hippocampus [F(2, 9) = 4.494, YKS: *P* < 0.05; KST: *P* < 0.05]. On the other hand, only YKS improved the phosphorylation of CREB [YKS: F(2, 9) = 4.435, YKS: *P* < 0.05; KST: *P* = 0.127].

## Discussion

Our previous studies have demonstrated that social isolation rearing of mice from early weaning induces behavioral abnormalities such as hyperactivity, reduced sociability, spatial attention deficit, and impaired fear memory performance. Considering the face and predictive validities of ISO mice, we have proposed that ISO from early weaning offers an animal model of comorbid developmental disorder with ADHD and ASD symptoms, and that the ISO model is useful for screening drugs for the treatment of such disorders [18, 20]. In this study, we investigated the therapeutic and preventive/delaying potentials of YKS and KST against ISO-induced behavioral and pharmacological abnormalities in order to elucidate the possible efficacy of Kampo medicines for the treatment of neurodevelopmental disorders. Our findings suggested that YKS and KST may be useful for the prevention or delaying of onset in developmental disorders.

In the experiments we first conducted to elucidate the therapeutic potentials of YKS and KST for treating ISO-induced behavioral abnormalities, the administration of YKS significantly improved impairments of attention-like behavior in the water finding test and context-dependent fear memory performance in the fear conditioning test, but not sociability in the 3-chamber test, whereas KST had no significant effects on these behavioral indices. This finding suggests that YKS can exert a therapeutic action against some symptoms related to attention deficit and memory impairment in the ISO model. In Kampo medicine, YKS is prescribed particularly to target neuropsychiatric symptoms in children, while KST is used to improve the initial symptoms of children with a headache and fever. Therefore, the differences in such utilization of YKS and KST are likely relevant to the differences in the therapeutic activity observed between YKS and KST. In previous studies, we demonstrated that acute administration of MPH ameliorated ISO-induced spatial attention deficits in the water finding test in part via central cholinergic systems, and that dysfunction of central cholinergic systems was involved in deficits of contextual and conditional fear memory caused by ISO from early weaning [19, 20]. Moreover, several lines of experimental evidence indicate that YKS is able to improve behaviors related to spatial cognitive dysfunction in animal models of dementia via central cholinergic systems [30]. Taken together, the present findings suggest that the ameliorative effects of YKS on spatial cognition-related behaviors in the water-finding and fear conditioning tests may be due to the distinctive pharmacological profiles of this Kampo medicine, which in part involves central cholinergic mechanisms.

It is of interest that in the experiments where the preventive/delaying potentials of YKS and KST were examined by administration in week 0 of the ISO period, both YKS and KST improved ISO-induced deficits in sociability- and attention-like behaviors as well as impairment of context-dependent fear memory. These findings indicate that both YKS and KST can exert preventive/delaying actions against behavioral abnormalities induced by ISO. We previously reported that MPH ameliorated sociability deficits caused by ISO, and that the effect of MPH was diminished by pre-treatment with SCH23390, a dopamine D_1_ receptor antagonist, indicating that sociability deficits in ISO mice are at least in part attributable to alterations of dopaminergic function in the brain [20]. However, in the present study, the preventive/delaying effect of YKS or KST on sociability deficits in ISO mice was unaltered by SCH23390, suggesting that, unlike MPH, a mechanism(s) independent of dopaminergic systems may be involved in the preventive/delaying effects of YKS and KST.

The present study also demonstrated that YKS and KST exhibited preventive/delaying effects on ISO-induced deficits of attention-like behavior in a manner reversed by scopolamine. This finding indicates the involvement of central cholinergic systems in the effects of these Kampo formulae, and supports at least the aforementioned therapeutic effect of YKS on ISO-induced deficits in attention-like behavior. This is supported by previous findings reported by Ouchi et al. [18]. They found that MPH amelioration of attention-like behavior in ISO animals could be reversed by scopolamine, suggesting the involvement of cholinergic dysfunction in impaired attention-like behavior. Several mechanisms may account for the preventive/delaying effects of YKS and KST. First, it is likely that the administration of YKS and KST during the ISO period facilitates the function of central cholinergic systems and thereby ameliorates attention-like behavior deficits in ISO animals. This possibility seems plausible for YKS rather than KST since, as discussed above, KST failed to exert therapeutic actions against ISO-induced behavioral abnormalities including impaired attention-like behavior. Second, both YKS and KST may be able to protect central cholinergic systems from dysfunction, which is induced from the early stages of ISO [18]. This mechanism is very likely to be implicated in the effect of KST, since KST ameliorated ISO-induced behavioral deficits when the administration was started from week 0, but not from week 2 after starting ISO. Moreover, chemical constituents of YKS and KST have been reported to have neuroprotective effects [33–36], suggesting that these compounds protect central cholinergic systems from ISO-induced dysfunction. This hypothesis is currently under investigation in our laboratory.

Importantly, the present neurochemical studies conducted according to the experimental schedule in Fig. 1b clearly revealed that daily administration of YKS and KST during the entire duration of ISO reversed the down-regulated expression of phosphorylated forms of CaMKII and CREB, neuroplasticity-related signaling proteins, in the hippocampus. We analyzed these proteins in the hippocampus because of a couple of reasons. First, synaptic plasticity-related signaling in the hippocampus is an important molecular biological basis of learning and memory including conditional fear memories [19, 37, 38]. Secondly, our previous study demonstrated that ISO of mice for 2 weeks after a weaning period impaired the consolidation process of contextual and auditory fear memories via causing dysfunction of hippocampal synaptic plasticity-related neuro-signaling, and that tacrine, an acetylcholinesterase inhibitor, ameliorated ISO-induced deficits in fear memories and hippocampal neuro-signaling function [19, 20]. Our previous findings have suggested that ISO-induced fear memory deficits and dysfunction of hippocampal synaptic plasticity-related neuro-signaling are in part due to impairment of central cholinergic systems, and that amelioration or prevention of cholinergic deficit contributes to the improvement of these impairments induced by ISO [20]. Considering the present data that daily administration of YKS and KST during the entire duration of the ISO period ameliorated not only contextual fear memory deficits, but also impairment of hippocampal neuro-signaling in ISO mice as well as tacrine, the present findings raise the possibility that YKS and KST may be able to prevent cholinergic systems from ISO-induced brain dysfunction via normalizing neuroplasticity-related hippocampal neuro-signaling, and thereby improve fear memory deficits. This idea is supported by previous reports that YKS ameliorated olfactory bulbectomy-induced deficits in spatial memory deficit, and that the effects were at least partly mediated by muscarinic receptor stimulation and the normalization of central cholinergic systems [30, 39].

One of the most interesting findings in this study is that not only YKS but also KST exerted significant preventive/delaying effect on ISO-induced behavioral abnormalities and hippocampal neuro-signaling deficits, because the clinical applications of KST significantly differ from those of YKS. The mechanism(s) and chemical constituent(s) involved in the actions of KST are still unclear. However, considering the fact that when administration of KST was conducted for almost a same period by the therapeutic procedure, KST did not affect ISO-induced behavioral abnormalities, it is very likely that KST may be able to block processes including epigenetic alteration, which is triggered early after starting ISO. Recent findings reported by Araki et al. have demonstrated that ISO induces epigenetic changes in promoter coding regions of the *GABA*_*B1a*_ *receptor* [40] and *srd5a1* [41], a gene coding for the biosynthetic enzyme neurosteroid allopregnanolone, which is down-regulated by ISO [16, 42]. Moreover, our preliminary study indicated that inhibition of allopregnanolone biosynthesis in the brain caused impairment of sociability behavior in mice in a manner reversible by systemic administration of allopregnanolone (unpublished data). Therefore, ISO mice may show behavior abnormalities through the epigenetic regulation, and it is possible that ISO-induced epigenetic alterations of the aforementioned genes and others are prevented by the administration of KST from an early stage of ISO. Further studies are needed to examine this possibility involved in the preventive/delaying effect of KST.

## Conclusion

In conclusion, the present studies demonstrated that daily administration of YKS and KST ameliorates ISO-induced impairments of sociability and spatial attention, as well as fear memory deficits, by restoring cholinergic and other neuron functions. Considering the neuropharmacological features of YKS and KST, it is likely that these Kampo formulae may offer a new class of preventive/delaying agents which can be used from an early stage of comorbid developmental disorders with ADHD and ASD symptoms, and that YKS is effective as a therapeutic agent to treat symptoms relevant to psychiatric comorbidity.

## Additional file

Additional file 1: A protocol to access and display the files of chemical profiling mass spectrometry data. (DOCX 15 kb)

## Abbreviations

ADHD: Attention deficit hyperactivity disorder
ASD: Autism spectrum disorder
GH: Group housing
ISO: Social isolation
KST: Keishito
MPH: Methylphenidate
PVDF: Polyvinylidene difluoride
SDS: Sodium dodecyl sulfate
SDS-PAGE: SDS-polyacrylamide gels
YKS: Yokukansan
