# Supplementary material for: Daily administration of yokukansan and keishito prevents social isolation-induced behavioral abnormalities and down-regulation of phosphorylation of neuroplasticity-related signaling molecules in mice
Source: BMC Complement Altern Med. 2017 Apr 4;17:195. doi: 10.1186/s12906-017-1710-7 (PMC5379572; doi:10.1186/s12906-017-1710-7)
Supplement: Additional file 1: — A protocol to access and display the files of chemical profiling mass spectrometry data. (DOCX 15 kb) [file 12906_2017_1710_MOESM1_ESM.docx]

**Additional file 1.** A protocol to access and display the files of chemical profiling mass spectrometry data

1.　Access the web page of Shimadzu Corporation (http://www.shimadzu.co.jp/aboutus/ms_r/masspp.html) and download the software supplied by Shimadzu Corporation (Mass++ (64-bit) 2.7.4).

2. Access the following web pages and download the files of mass spectrometry data of YKS and KST.

YKS: http://dentomed.u-toyama.ac.jp/en/information_on_experimental_kampo_extracts/yokukansan-2016-KM-extract/EXP081002

KST: http://dentomed.u-toyama.ac.jp/en/information_on_experimental_kampo_extracts/keishito-2016-KM-extract/EXP010002

3. Display raw data of YKS and KST using the downloaded software.
